# A Novel Reagent for Radioiodine Labeling of New Chemical Entities (NCEs) and Biomolecules

**DOI:** 10.3390/molecules26144344

**Published:** 2021-07-18

**Authors:** Krishan Kumar, Karen Woolum

**Affiliations:** Laboratory for Translational Research in Imaging Pharmaceuticals, The Wright Center of Innovation in Biomedical Imaging, Department of Radiology, The Ohio State University, Columbus, OH 43212, USA; Karen.Woolum@osumc.edu

**Keywords:** radioiodine labeling, radioiodination, radiotracers, biomolecules, peptides, proteins, monoclonal antibodies, radiopharmaceuticals, imaging pharmaceuticals, ^123,124,125,131^I-labeled molecules and biomolecules

## Abstract

Radioiodine labeling of peptides and proteins is routinely performed by using various oxidizing agents such as Chloramine T, Iodobeads, and Iodogen reagent and radioactive iodide (I^−^), although some other oxidizing agents were also investigated. The main objective of the present study was to develop and test a novel reagent, inorganic monochloramine (NH_2_Cl), for radioiodine labeling of new chemical entities and biomolecules which is cost-effective, easy to make and handle, and is selective to label amino acids, peptides, and proteins. The data presented in this report demonstrate that the yields of the non-radioactive iodine labeling reactions using monochloramine are >70% for an amino acid (tyrosine) and a cyclic peptide (cyclo Arg-Gly-Asp-d-Tyr-Lys, cRGDyK). No evidence of the formation of *N*-chloro derivatives in cRGDyK was observed, suggesting that the reagent is selective in iodinating the tyrosine residue in the biomolecules. The method was successfully translated into radioiodine labeling of amino acid, a peptide, and a protein, Bovine Serum Albumin (BSA).

## 1. Introduction

Radioiodine labeling (Radioiodination) of molecules, using radioactive iodide, was first established a long time ago when ^131^I isotope of iodide was used for the labeling of polyclonal anti-kidney serum [1]. Since then, the radioiodine labeling technique is being used in the evaluation of New Chemical Entities (NCEs) and small and large biomolecules for their biological and medical applications. Four isotopes of iodine (^123^I, ^124^I, ^125^I, and ^131^I) are used routinely for radioiodine labeling of NCEs and biomolecules, depending on the intended application. For example, ^125^I radionuclide, with a long half-life of 59.9 days, is used for radioiodine labeling of a molecule or a biomolecule for its pharmacokinetics, metabolism, and biodistribution studies. On the other hand, ^123^I, with a half-life of 13.1 h, and ^124^I, with a half-life of 4.17 d, are used for the evaluation of a molecule or a biomolecule for its SPECT (Single Photon Emission Computed Tomography) and PET (Positron Emission Tomography) imaging applications, respectively. ^131^I radionuclide, a beta particle emitter, is used for the therapeutic application of a radiolabeled molecule or biomolecule.

For direct radioiodine labeling of a molecule or a biomolecule, the presence of an aromatic moiety, tyrosine or histidine is required. The primary site of the iodine addition is tyrosine amino acid residue in NCEs or biomolecules; however, if the pH exceeds 8.5, the secondary site on the imidazole ring of histidine is preferred. The tyrosine moiety can be labeled twice, giving a mixture of mono and di-iodinated species. The formation of di- iodinated tyrosine is faster than the mono-iodinated tyrosyl moiety. Several reports related to the methods and reagents of radioiodine labeling of molecules and biomolecules have been published in the past [2,3]. Every effort must be made, regardless of the application of the radioiodine-labeled molecule or biomolecule, to maintain immunoreactivity and high molar activity of the biomolecule after labeling and purification.

Radioiodine labeling of a molecule or a biomolecule, peptides and proteins, involves an oxidizing agent and an iodide radionuclide which is usually available as Sodium Iodide (NaI) in neutral or basic aqueous solutions. A large number of inorganic and organic oxidizing reagents have been used, in the past, for radioiodine labeling of biomolecules, peptides and proteins. This includes I_2_ [1], sodium hypochlorite [4], nitrous acid [5,6], ammonium persulfate [7], hydrogen peroxide [8], ferric sulfate [9], iodate [10], iodine monochloride [11,12], hypochlorite/hypochlorous acid [13], IBPy_2_BF_4_ [14], Penta-*O*-Acetyl-*N*-Chloro-*N*-Methylglucamine [15,16], *N*-chloro derivatives of secondary amines [17], *N*-chloromorpholine [18], Chloramine-T [19,20], iodobeads [21], lodogen [22,23], and Iodogen reagent coated on the bottom of tubes (commonly known as the Iodination tubes) [24,25]. Some enzymes are known for catalyzing the mild oxidation of iodide for radioiodine labeling of tyrosine, and to some extent histidine also, in proteins [26,27]. If it is not possible to radioiodine label proteins by direct electrophilic addition to tyrosine and histidine residues, a most common alternative approach (indirect radioiodine labeling method) is using a prosthetic group for radioiodine labeling of NCEs and biomolecules [28,29].

**Figure 1:**
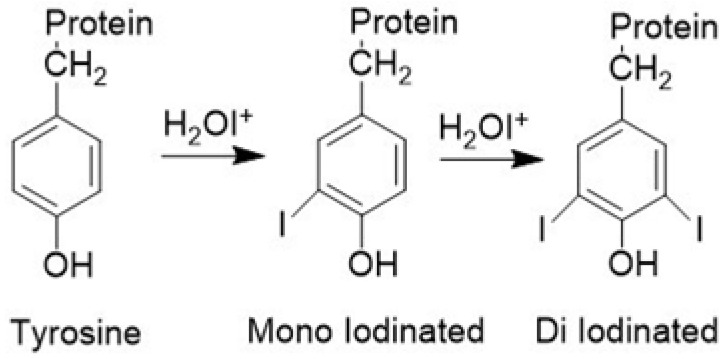
Radioiodine labeling scheme for tyrosine residue in peptides and proteins.

Iodination tubes, the most popular and convenient, are used routinely in research laboratories. Iodogen in the iodination tube, like Chloramine T, converts I^−^ to I^+^ (H_2_OI^+^) or ICl followed by an electrophilic substitution reaction on the aromatic moiety in peptides and proteins (Figure 1), forming mono- and di-iodinated tyrosyl residues [30].

Iodogen, like Chloramine-T, has the potential to over radioiodine label or sometimes damage and form an *N*-chloro derivative of a lysine-residue in peptide and proteins [8]. For this reason, *N*-chloroderivatives of secondary amines with low oxidation potentials were tested as potential radio iodine labeling agents [17,18]. These *N*-chloroderivatives were prepared fresh and used immediately due to the instability of these materials.

The main objective of the present study was to evaluate an agent which is cost-effective, easy to make and handle, contains active chlorine for the conversion of I^−^ to I^+^, and is selective for non-radioactive iodine/radioiodine labeling of tyrosine containing peptides and proteins, with iodine rather than forming *N*-chloro derivatives of amino acids in peptides and proteins containing primary amines. For this purpose, we selected an inorganic monochloramine (NH_2_Cl) due to (1) easy in situ formation from the reaction of ammonium hydroxide and sodium hypochlorite, (2) the reagents, ammonium hydroxide and sodium hypochlorite, are inexpensive, (3) the redox potential of monochloramine is lower than Chloramine-T and, consequently, NH_2_Cl may be less damaging than Chloramine-T to biomolecules [31], (4) oxidation of I^−^ to I^+^ by monochloramine occurs via chlorine atom transfer and the rate of the oxidation reaction is very fast under neutral pH conditions [32], and (5) the routinely used oxidizing agents, chloramine T, iodobeads, and iodogen, probably, follow the same mechanism as NH_2_Cl. In this present work, we have conducted a systematic nonradioactive iodine/radioiodine labeling (^127^I/^125^I labeling) study of an amino acid, tyrosine, a cyclic peptide, cRGDyK (cyclo Arg-Gly-Asp-d-Tyr-Lys), and a protein, Bovine Serum Albumin (BSA), containing tyrosine and histidine residues, for the development of a novel reagent for non-radioactive iodine and radioiodine labeling.

A cyclic peptide, cRGDyK, selected in this study, is interesting in many ways: (1) monomeric, dimeric, and tetrameric cyclic RGD peptides have shown binding affinity to α_v_β_3_ integrin, an angiogenic biomarker which is overexpressed in the endothelium of most solid tumors. (2) Several radiolabeled cyclic RGD peptides have been investigated as potential radiotracers for angiogenesis imaging [33,34]. (3) Presence of lysine amino group in cRGDyK provides additional possibilities for dual probes development, i.e., conjugating with dyes to produce optical probes. (4) Transfer of chlorine from NH_2_Cl to nitrogen in amines, amino acids, and peptides is a thermodynamically favorable reaction [35]. NH_2_Cl mediated labeling of cRGDyK, which contains lysine along with tyrosine residue, will also demonstrate the selectivity of the non-radioactive iodine/radioiodine labeling procedure.

The data presented in this report demonstrate that the yields of the non-radioactive iodine labeling reaction using monochloramine are high for an amino acid (tyrosine) and a cyclic peptide (cyclo Arg-Gly-Asp-d-Tyr-Lys, cRGDyK). No evidence of the formation of *N*-chloro derivative in cRGDyK was observed, suggesting that the reagent is selective in iodinating the tyrosine residue in the biomolecules. The method was successfully translated into radioiodine labeling of an amino acid, a peptide, and a protein, Bovine Serum Albumin (BSA).

## 2. Results and Discussion

### 2.1. Preparation and Characterization of Monochloramine

Monochloramine (NH_2_Cl) was prepared fresh daily by mixing 10% to 20% excess ammonium hydroxide and sodium hypochlorite at pH 10 [30]. The concentrations of sodium hypochlorite stock solutions and the prepared monochloramine samples were determined from the measurement of their absorbances and molar extinction coefficients at the absorbance maxima [9]. The absorption maximum (nm) and the molar extinction coefficient (M^−1^cm^−1^) for sodium hypochlorite and monochloramine are 292, 350 and 243, 461, respectively [30]. The rate of formation of monochloramine from the reaction of hypochlorite and ammonia is fast and complete. The amount of hydrazine from the Raschig Synthesis and nitrogen is expected to be low due to the limited amount of excess ammonia. Raschig synthesis usually requires a large amount of excess ammonia [36]. Freshly prepared monochloramine under basic conditions was used immediately to avoid any formation of di or trichloramines from NH_2_Cl by the disproportionation reactions.

### 2.2. Non-Radioactive Iodine Labeling of Tyrosine and cRGDyK

In several non-radioactive iodine labeling experiments with tyrosine, known amounts of tyrosine (0.8–2.2 μmole), sodium iodide (^127^INa) solution (0.9–2.35 μmole), and monochloramine (1–3 μmole) were reacted in a small glass vial or Eppendorf tube containing 0.1 mL sodium phosphate buffer (0.1 M, pH 7.4). The reaction mixture was incubated at room temperature for 30 min. At the end of the incubation time, the reaction was quenched by adding the reducing agent, a freshly prepared sodium metabisulfite solution. The reaction mixture was analyzed using a gradient Reversed-Phase High-Performance Liquid Chromatography (RP-HPLC) method.

**Figure 2:**
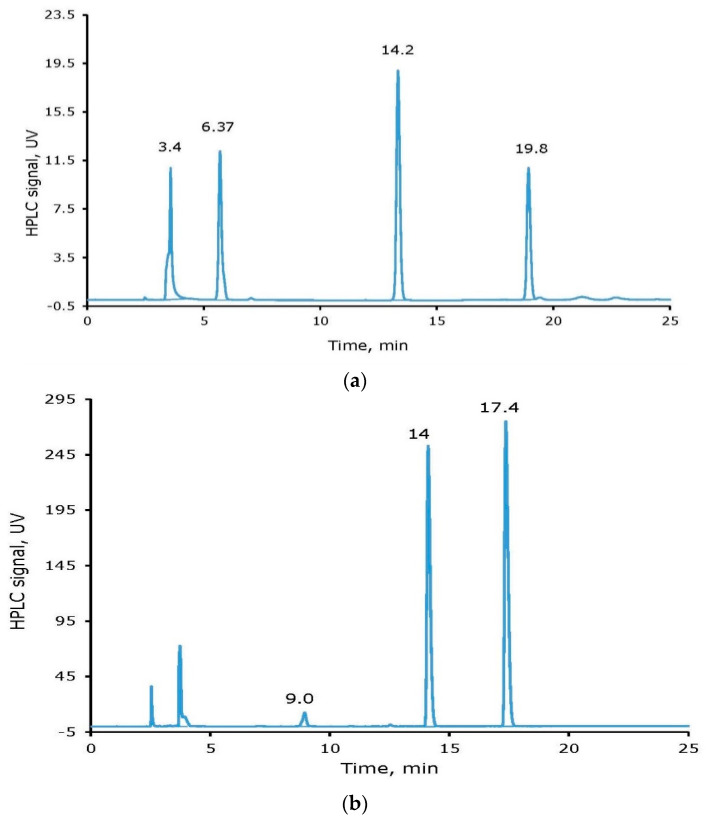
HPLC chromatograms of the reaction mixtures of non-radioactive iodine labeling of tyrosine (**a**) and cyclo Arg-Gly-Asp-d-Tyr-Lys (cRGDyK) (**b**).

Four peaks (Retention Times, RT in min, given in parenthesis), unreacted Iodide (3.4) and tyrosine (6.4), mono-iodinated tyrosine (I-Tyr, 14.2,) and di-iodinated tyrosine (I_2_-Tyr, 19.8), with a variable ratio dependent on the reaction conditions, were observed. The peak for unreacted iodide is in the form of oxidized iodide. The yield of the non-radioactive iodine labeling reaction was calculated based on the limiting reagent, tyrosine, as high as >85%. Figure 2 (top) shows a representative HPLC chromatogram for a reaction mixture in which 0.6 µmole of tyrosine, 0.52 µmole sodium iodide, and 0.55 µmole NH_2_Cl were used. The percentage peak areas observed were 24.5, 20.8, 34.5, and 20.11 for unreacted sodium iodide, unreacted tyrosine, I-tyrosine, and I_2_-tyrosine, respectively. The calculated yield for the formation of iodinated tyrosine is ~72%.

Similarly, for non-radioactive iodine labeling of a cyclic peptide, 0.4 µmole of cRGDyK, 0.47 µmole sodium iodide, and 0.4 µmole NH_2_Cl, were reacted in an Eppendorf tube for 30 min. At the end of the reaction, 0.58 µmole freshly prepared sodium metabisulfite was added. Four peaks (Retention Times, RT in min, and percentage peak areas given in parenthesis), unreacted Iodide (3.7, 1.85) and cRGDyK (9.0, 2.4), mono-iodinated-cRGDyK (I-cRGDyK, 14.0, 44.0) and di-iodinated –cRGDyK (I_2_-cRGDyK, 17.4, 52. 0), were observed (Figure 2, bottom). There was also a small solvent front peak that was not integrated for further calculations. The yield of the non-radioactive iodine labeling reaction was calculated based on the limiting reagent, cRGDyK, as 97%. Additional, non-radioactive iodine labeling experiments with cRGDyK were performed in 1:1:1 (0.5 µmole scale cRGDyK) and 2:1:1 (1 µmole scale cRGDyK) mole ratios of cRGDyK:I:NH_2_Cl. The yields of the formation of the iodinated cRGDyK (the sum of I-cRGDyK and I_2_-cRGDyK) were 77% and 71% for the 1:1:1 and 2:1:1 reaction mixture, respectively. There is always a mixture of mono- and di-iodinated tyrosine and cRGDyK; however, the ratio of the two species is dependent on the concentrations of the reactants and reaction conditions.

**Figure 3:**
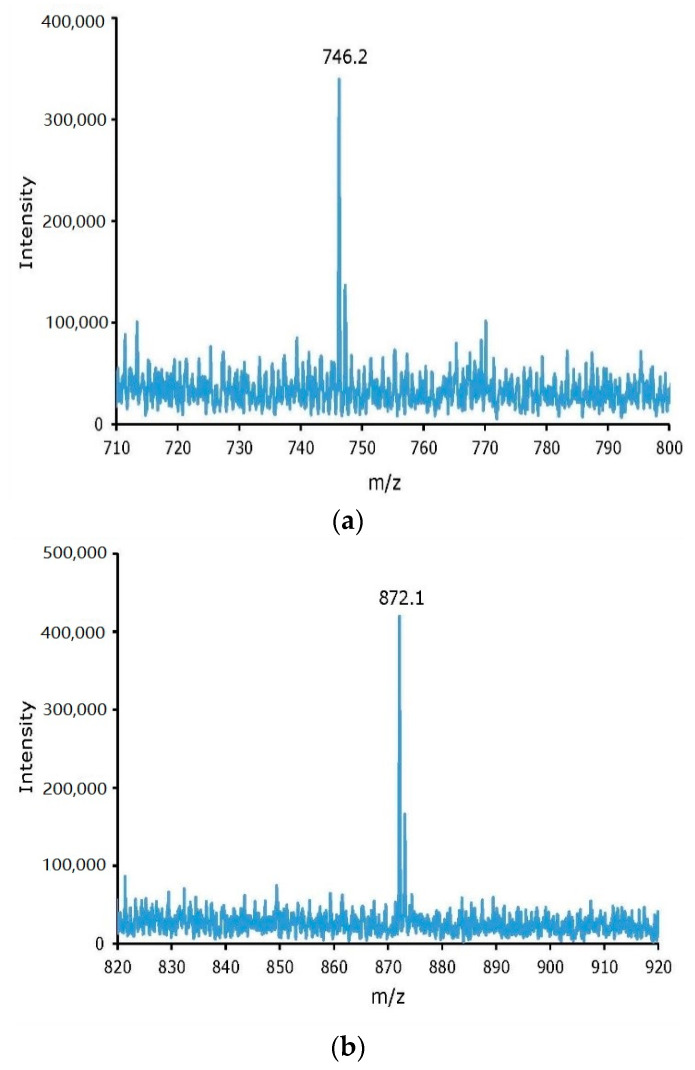
ESI mass spectra of two HPLC chromatogram peaks collected from the non-radioactive iodine labeling reaction mixture of cyclo Arg-Gly-Asp-d-Tyr-Lys (cRGDyK), I-cRGDyK (**a**) and I2-cRGDyK (**b**).

The crude non-radioactive iodine labeling reaction mixtures of tyrosine and cRGDyK were purified by an RP Sep-Pak cartridge method, to remove unreacted iodide, and RP-HPLC purification method, to remove unreacted tyrosine or cRGDyK. The two RP-HPLC peaks, at 14.2 and 19.8 min retention times from the non-radioactive iodine labeling of tyrosine, were collected, concentrated, and confirmed by Electrospray Ionization (ESI) mass spectra as mono- and di-iodinated tyrosine with *m*/*e* peaks, for (m + H)^+^, at 306.8 (calculated 307.09) and 433.8 (calculated 433.9), respectively. Similarly, the identity of I- cRGDyK and I_2_- cRGDyK was confirmed by ESI mass spectra after collecting the 14 and 17.4 min peaks from the non-radioactive iodine labeling reaction mixture of cRGDyK. The *m*/*e* peaks, for (m + H)^+^, were observed as 746.2 (calculated 746.68) and 872.1 (calculated (872.58) for I- cRGDyK and I_2_- cRGDyK, respectively. As shown in Figure 3, the ESI mass spectrum did not show any evidence of the formation of the *N*-chloro derivative of cRGDyK. On the contrary, non-radioactive iodine labeling of cRGDyK using the Iodogen method showed evidence of *N*-chlorination of the lysine residue in the cyclic peptide [13].

### 2.3. Radioiodine Labeling of Tyrosine and Cyclo Arg-Gly-Asp-d-Tyr-Lys (cRGDyK)

The non-radioactive iodine labeling reaction protocols were translated into radioiodine labeling of tyrosine and cRGDyK. For radioiodine labeling of tyrosine and cRGDyK, 0.64 μmole of tyrosine, 212 μCi ^125^I, and 0.45 μmole NH_2_Cl and 0.2 μmole of cRGDyK, 198 μCi ^125^I, and 0.2 μmole NH_2_Cl, respectively, were mixed in 0.1 mL sodium phosphate buffer (0.1 M, pH 7.0). The reaction mixtures were incubated at room temperature for 30 min. At the end of the incubation time, the radioiodine labeling reactions were quenched by the addition of a freshly prepared sodium metabisulfite solution. From the RP-HPLC (the method conditions given in the experimental section) analysis of the crude material, it was observed that the radioiodine incorporation into tyrosine and cRGDyK was 83.2% and ~99%, respectively.

**Figure 4:**
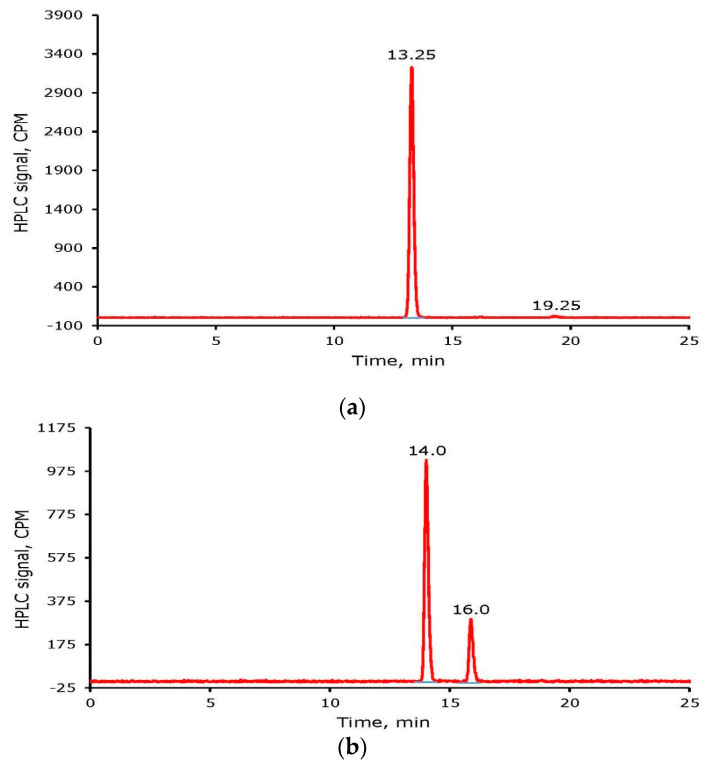
HPLC Chromatogram of radioiodine labeled tyrosine (**a**) and cyclo Arg-Gly-Asp-d-Tyr-Lys (cRGDyK) (**b**) after Sep-Pak purification.

Purification of the reaction mixture of a radioiodine-labeled tyrosine or cRGDyK was accomplished initially by using the Sep-Pak method. Several fractions containing approximately 10 drops were collected and counted for radioactivity during Sep-Pak purification. All major fractions were combined and concentrated to near dryness under a stream of nitrogen at room temperature. The final product was reconstituted in water or Phosphate Buffer Saline (PBS). Seventy to eighty percent of the radioiodine-labeled materials were recovered after Sep-Pak purification. Figure 4 shows HPLC chromatograms of radioiodine labeled tyrosine and cRGDyK after Sep-Pak purification. Due to the non-carrier-added nature of the radioiodine-labeling reactions, the formation of ^125^I_2_-tyrosine and ^125^I_2_-cRGDyK is low. For example, the percentages (given in the parenthesis) are: ^125^I-tyrosine (96), ^125^I_2_-tyrosine (4), ^125^I-cRGdyK (93.4), and ^125^I_2_-cRGDyK (6.6). Higher percentages of di-iodinated tyrosine and cRGDyK were seen in non-radioactive iodine labeling experiments.

Further HPLC purification was performed to separate mono and di- radioiodine-labeled tyrosine or cRGDyK. HPLC fractions were collected, concentrated, and analyzed. The fractions of the mixture of mono- and di- ^125^I-labeled tyrosine and cRGDyK were collected. The calculated recovery (sum of the two) after purification was 65% to 75%.

**Figure 5:**
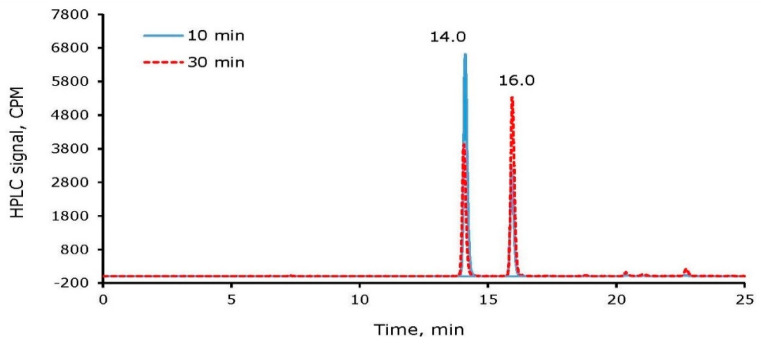
The percentage of ^125^I-cRGDyK and ^125^I_2_-cRGDyK after incubation of the reaction mixture for 10 and 30 min, respectively.

To optimize the reaction time for radioiodine labeling of tyrosine and cRGDyK, a mixture of 0.16 µmole of cRGDyK, 356 µCi ^125^I, and 0.22 µmole of NH_2_Cl were reacted. An aliquot of the reaction mixture was analyzed at 10 min and 30 min after quenching the reaction. From the time-dependent radioiodine incorporation into cRGDyK, it was concluded that 10 min of incubation of the reaction mixture is sufficient for the completion of radioiodine labeling of the tyrosine residue in cRGDyK. Longer period incubation converted mono-iodinated cRGDyK to an increased percentage of di-iodinated species. For example, the ratios of ^125^I-cRGDyK:^125^I_2_-cRGDyK were observed as ~67:33 and ~42:58 after 10 and 30 min incubation, respectively (Figure 5). The amount of ^125^I_2_-cRGDyk at 30 min in this study is higher than the study above, possibly due to the higher amount of ^125^I (giving higher ^125^I radioactivity/mass ratio) used in this study.

### 2.4. Radioiodine Labeling of Bovine Serum Albumin (BSA)

For radioiodine labeling of BSA, 50 μg (50 μL of 1 mg/mL) of the BSA solution was transferred into an Eppendorf tube containing 100 μL of sodium phosphate buffer (0.1 M pH 7.4). Carrier-free ^125^INa (~93 µCi) followed by monochloramine (200 μL of 4.14 mM, 0.8 μmole) were added to the tube and incubated at room temperature for 30 min. The reaction was quenched by the addition of sodium metabisulfite. The crude reaction mixture was purified using a PD-10 column by loading radioiodine-labeled BSA onto the column and eluting with PBS. Small fractions were collected into pre-labeled microcentrifuge tubes. The fractions containing most of the activity were pooled and counted for radioactivity.

The incorporation of radioiodine into BSA (i.e., yield) was calculated as 82.4% from the ratio of radioactivity recovered from the elution of the PD-10 and the amount of radioactivity taken initially for radioiodine labeling of BSA. The PD-10 column purified radioiodine-labeled BSA was analyzed for radiochemical purity (RCP), and free radioiodide by using a Paper Chromatography and a Size-Exclusion High-Performance Liquid Chromatography (SEC-HPLC) methods. In the paper chromatography method, the radioiodine labeled BSA precipitates at the origin in the 85:15 methanol: water developing phase and radioiodide moves to the solvent front. The percent RCP and free radioiodide of radioiodine-labeled BSA were calculated from the measured CPM, by Capintec well counter, of the bottom half and top half of the paper strip, respectively (Equations (1) and (2)).

% RCP of radioiodine-labeled BSA = (Counts on the bottom half/Total
counts from top and the bottom halves) × 100
(1)

% Free radioiodide = (Counts on the top half/Total counts from top and the bottom halves) × 100(2)

**Figure 6:**
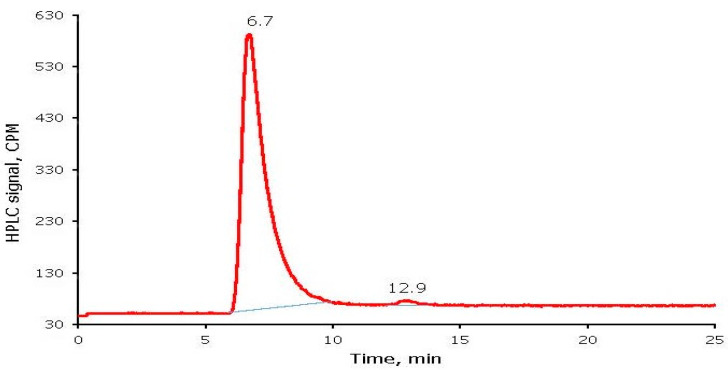
HPLC chromatogram of radioiodine labeled Bovine Serum Albumin (BSA).

For a representative radioiodine-labeled BSA sample, top and bottom halves had 4.58 and 212.2 kCPM, respectively. The RCP and free radioiodide of radioiodine labeled BSA were calculated from the Paper Chromatography method as 97.9% and 2.1%, respectively. An SEC-HPLC chromatogram for the same radioiodine labeled BSA sample is shown in Figure 6. Radioiodine labeled BSA and free radioiodide eluted at 6.7 and 12.9 min, respectively. Consistent with the Paper Chromatography results, the SEC-HPLC showed RCP of radioiodine labeled BSA as 98.7% with free radioiodide as 1.3%.

### 2.5. Comparison of Monochloramine with Other Oxidizing Agents

Like other oxidizing agents, Chloramine-T, iodobeads, Iodogen, etc., monochloramine is a useful and effective oxidizing agent for radioiodine labeling of amino acids, peptides, and proteins. Monochloramine is easy to prepare and handle and is cost-effective due to the use of inexpensive reagents, ammonium hydroxide and sodium hypochlorite. The yields of radioiodine labeling and molar activities using monochloramine and other oxidizing agents are expected to be comparable. Using all oxidizing agents requires the purification steps to remove unreacted radioiodide and the amino acid or peptide. Radioiodine labeling using monochloramine is selective, i.e., no evidence of the formation of *N*-chloro derivative of cRGDyK while previous studies, using Iodogen, have shown the formation of *N*-chloroderivative of cRGDyK [13]. The rate of radioiodine labeling, using NH_2_Cl, is faster, such as Chloramine-T, as these are solution–solution phase reactions. Chloramine-T and other oxidizing agents have shown damage to proteins under certain conditions [8]. Similarly, NH_2_Cl also has the potential to oxidize –SH groups in proteins. However, the lower redox potential of NH_2_Cl than Chloramine-T and faster rates of oxidation of I^−^ to I^+^ than oxidation of –SH groups by NH_2_Cl makes it less likely [31,37]. Like other oxidizing agents, radioiodine labeling conditions, i.e., amount of NH_2_Cl and incubation time, must be optimized before routine radiolabeling of proteins using inorganic monochloramine.

## 3. Materials and Methods

### 3.1. General

All chemicals and reagents, tyrosine and BSA (Sigma-Aldrich, St. Louis, MO, USA), cRGDyK (Peptide International, Louisville, KY, USA), sodium iodide (Acros, Sommerville, NJ, USA), ammonium hydroxide and sodium hypochlorite (Fisher Scientific, Fair Lawn, NJ, USA), and sodium bisulfite (Sigma-Aldrich) were used as received. Sodium monobasic phosphate, sodium dibasic phosphate, sodium hydroxide, hydrochloric acid and sodium chloride (all from Fisher Scientific) were used for buffer and mobile phase preparations and pH and ionic strength control. Gibco 1X PBS (pH 7.4) buffer was supplied by Fisher Scientific. Crude reaction mixtures from the non-radioactive iodine/radioiodine labeling reactions of tyrosine and cRGDyK and BSA were purified to remove unreacted non-radioactive/radioactive sodium iodide by using a Reversed-Phase Sep-Pak C_18_ Light cartridge (Waters, Milford, MA, USA) and a PD-10 column (GE Healthcare, Chicago, IL, USA), respectively. For radioiodine labeling experiments, ^125^INa was purchased from Perkin Elmer (Shelton, CT, USA).

### 3.2. Chemistry

For the preparation of the tyrosine stock solution, it was necessary to add diluted HCl to lower the pH ~5 initially for its solubilization followed by solution pH adjustment to 7 by the addition of a sodium phosphate buffer. Sodium iodide and cRGDyK solutions were prepared in water. Since the ^125^I Na sample is supplied in a 0.1 N sodium hydroxide base solution, it was occasionally necessary to adjust the pH of the solution to ~7 by the addition of a small amount of hydrochloric acid. Monochloramine (NH_2_Cl) was prepared fresh daily as described elsewhere by mixing 10% to 20% excess ammonium hydroxide and sodium hypochlorite at a pH ~10 [30]. The final pH of the monochloramine solution was adjusted to ~8 with HCl. The concentration of sodium hypochlorite stock solution and monochloramine sample was determined spectrophotometrically [9].

### 3.3. Analytics

An Agilent 8453 model spectrophotometer was used for all UV/Vis spectral and absorbance measurements. A Capintec dose calibrator Model CRC-R (Capintec, Ramsey, NJ, USA) was used for the determination of radioactivity amounts in the ^125^INa source and the radioiodine-labeled materials. Agilent model 1100 HPLC systems (Agilent, Wilmington, DE, USA) were used for purification and analysis of non-radioactive iodine- and radioiodine-labeled tyrosine, cRGDyK, and BSA samples. These systems consisted of quaternary pumps, degasser, temperature-controlled column compartment, auto-injector, and multi-wavelength/diode array detectors and control by Agilent’s Chem Station or Lab Logics’ (Sheffield, UK) Laura software. For detection of radioiodine labeled materials, a Flow Scintillation Analyzer (FSA 150) from Perkin Elmer or a Flow Ram from Lab Logic Systems was used. A Capintec well counter model CRC-25W was used for the analysis of paper chromatography samples. ESI mass spectral analysis was used for the characterization of the nonradioactive-iodine labeled samples of tyrosine and cRGDyK. A Bruker amazon ETD mass spectrometer at Campus Chemical Instrument Center (CCIC) Mass Spectrometry and Proteomics Facility at The Ohio State University (OSU) was used.

### 3.4. Radiochemistry

In a typical non-radioactive iodine/radioiodine labeling experiment, known amounts of tyrosine or cRGDyK solution and ^127^INa or ^125^INa were mixed in a vial or Eppendorf tube containing 0.1–0.5 mL sodium phosphate buffer (0.1 M, pH 7.4). A known amount of monochloramine was added to the vial. The reaction mixture was agitated and mixed after the addition of each reagent by using a pipette, and incubated at room temperature for the desired time. At the end of the incubation period, the non-radioactive iodine or radioiodine labeling reaction was quenched by the addition of an excess of a freshly-prepared reducing agent, sodium metabisulfite. Purification of the reaction mixture of the non-radioactive iodine or the radioiodine labeled tyrosine or cRGDyK was accomplished in two steps. An RP-Sep-Pak C_18_ Light cartridge was used to remove any unreacted sodium iodide or ^125^I Na, followed by an RP-HPLC purification method to remove any unlabeled or unreacted tyrosine or cRGDyK.

The Sep-Pak purification method involved conditioning the Sep-Pak C_18_ Light cartridge with 3 mL of ethanol, washing with 3 mL water, loading of the crude material, washing with 1.5 mL water, followed by elution with 100 µL portions of 0.5 mL 100% ethanol. All major fractions were combined and concentrated to near dryness under a stream of nitrogen at room temperature. The final product was reconstituted in water or Phosphate Buffer Saline (PBS). The semi-purified mixture was analyzed and further purified by an RP-HPLC method involving a Zorbax C18 5 µm, 4.6 × 250 mm column, a flow rate of 1 mL/min, a UV detection at λ = 280 nm, a radioisotope detector, and a gradient mobile phase. The following gradient of water containing 0.1% TFA (A) and acetonitrile containing 0.1% TFA (B) was used: 95% A and 5% B initially, ramping the concentration of B to 25% in 20 min and then keeping it at 25% for 4 min. The concentration of B was brought down to 5% next in one min and kept up to 30 min.

For radioiodine labeling of BSA, a known amount of BSA was transferred into an Eppendorf tube containing 100 μL of sodium phosphate buffer (0.1 M pH 7.4). Carrier-free ^125^INa followed by monochloramine in sodium phosphate buffer were added to the tube. The reaction mixture was agitated and mixed with the pipette and incubated at room temperature for the desired time. The reaction was quenched by the addition of sodium metabisulfite in excess. The crude reaction mixture was purified using a PD-10 column. For purification of radioiodine-labeled BSA, the reaction mixture was loaded onto the conditioned column and eluted with PBS. Small fractions were collected into pre-labeled microcentrifuge tubes. The fractions containing most of the activity were pooled and counted for radioactivity.

The radioiodine-labeled BSA was analyzed by two methods, paper chromatography and SEC-HPLC. The Paper Chromatography method involved a 3 MM cellulose chromatography paper strip and an 85:15 methanol: water mixture as a developing solution. After the paper strip was developed and allowed to dry, the strip was then cut into two half pieces in the middle. Each piece of the strip was counted in a Capintec well counter. The Size-Exclusion HPLC method involved an Agilent SEC-3 100 Å column (4.6 × 300 mm), 150 mM sodium phosphate buffer pH 7.0, a flow rate of 0.35 mL/min, UV detection at λ = 280 nm, and a radioisotope detector.

## 4. Conclusions

The use of simple inorganic chloramine, NH_2_Cl, for non-radioactive and radioiodine labeling of a tyrosine residue in NCEs and biomolecules has been demonstrated in this present work. The non-radioactive iodine labeling method is selective, i.e., no evidence of the formation of *N*-chloro derivative, and gives a high yield, >70%. The method was successfully translated for radioiodine labeling of biomolecules. As seen in the case of Chloramine-T and other oxidizing agents, NH_2_Cl also has the potential to oxidize –SH groups in proteins [31,37]. However, the lower redox potential of NH_2_Cl than Chloramine-T and faster rates of oxidation of I^−^ to I^+^ than oxidation of –SH groups by NH_2_Cl makes it less likely.

## Data Availability

The data are included within the manuscript.

## Sample Availability

Samples of the compounds are not available from the authors.
